# Quality of life among people with mental illness attending a psychiatric outpatient clinic in Ethiopia: a structural equation model

**DOI:** 10.3389/fpsyt.2024.1407588

**Published:** 2024-08-12

**Authors:** Wondale Getinet Alemu, Lillian Mwanri, Clemence Due, Telake Azale, Anna Ziersch

**Affiliations:** ^1^ College of Medicine and Public Health, Flinders Health and Medical Research Institute, Flinders University, Adelaide, SA, Australia; ^2^ Department of Psychiatry, College of Medicine and Health Sciences, University of Gondar, Gondar, Ethiopia; ^3^ Research Centre for Public Health, Equity, and Human Flourishing, Torrens University Australia, Adelaide, SA, Australia; ^4^ School of Psychology, The University of Adelaide, Adelaide, SA, Australia; ^5^ Institute of Public Health, College of Medicine and Health Sciences, University of Gondar, Gondar, Ethiopia

**Keywords:** quality of life, mental wellbeing, mental illness, mental disorder, Ethiopia

## Abstract

**Background:**

Mental illness is one of the most severe, chronic, and disabling public health problems that affects patients’ Quality of life (QoL). Improving the QoL for people with mental illness is one of the most critical steps in stopping disease progression and avoiding complications of mental illness. Therefore, we aimed to assess the QoL and its determinants in patients with mental illness in outpatient clinics in Northwest Ethiopia in 2023.

**Methods:**

A facility-based cross-sectional study was conducted among people with mental illness in an outpatient clinic in Ethiopia. The sampling interval was decided by dividing the total study participants who had a follow-up appointment during the data collection period (2400), by the total sample size 638, with the starting point selected by lottery method. The interviewer-administered WHOQOL BREF-26 tool was used to measure the quality of life (QoL) of people with mental illness. The domains of QoL were identified, and indirect and direct effects of variables were calculated using structural equation modelling with SPSS-28 and Amos-28 software. A p-value of < 0.05 and a 95% CI were used to evaluate statistical significance.

**Results:**

A total of 636 (99.7%) participants agreed to participate and completed the data collection. The mean score of overall QoL of people with mental illness in the outpatient clinic was 49.6 ± 10 Sd. The highest QoL was found in the physical health domain (50.67 **±** 9.5 Sd), and the lowest mean QoL was found in the psychological health domain (48.41 ± 10 Sd). Rural residence, drug nonadherence, suicidal ideation, not getting counselling, moderate or severe subjective severity, family does not participate in patient care and a family history of mental illness had an indirect negative effect on QoL. Alcohol use and psychological health domain had direct positive effect on QoL. Furthermore, objective severity of illness, having low self-esteem, and having history of mental illness in the family had both direct and indirect effect on QoL. Furthermore, sociodemographic factors (rural residence, illiterate educational status, not married marital status), social support-related factors (poor self-esteem, family not participating in patient care), substance use factors (alcohol use, tobacco use) and clinical factors (high objective and subjective severity of illness, not getting counselling, suicidal ideation, higher number of episodes, comorbid illness, family history of mental illness, poor drug adherence) directly and indirectly affected QoL.

**Conclusions:**

In this study, the QoL of people with mental illness was poor, with the psychological health domain the most affected. Sociodemographic factors, social support-related factors, drug use factors, and clinical factors, directly and indirectly affected QoL through the mediator variables of physical health domains, psychological health domains, social relation health domains, and environmental health domains. In order to improve the QoL of people with mental illnesses, we recommend that emphasis be given to addressing the QoL of those with mental illness, including the development of policy and practice responses that address the above identified factors.

## Introduction

Mental illnesses are a global public health problem (1–3), a scourge that does not discriminate against populations. Evidence from the global burden of disease (GBD) 2019 indicated that there has been no reduction since 1990, and mental illness continues to be among the top ten leading causes of disease worldwide (4). Many of the world’s population have mental illness (5). The World Health Organization (WHO) indicates that 4% of the adult population worldwide is affected by severe mental illness (6), and 4.4% of African adults are affected (7). The WHO report on the African Region indicated that in 1990, 12.41% of the population had mental illnesses, with only a slight reduction of this figure to 12.21% in 2019 (8). Estimates for mental illness in Ethiopia indicate prevalence rates of 0.48 and 0.2 for schizophrenia and bipolar disorder respectively (9) and a WHO report shows that 4.7% & 3.3% people in Ethiopia are estimated to suffer from depression and anxiety, respectively (10).

Apart from heart disease, recent studies indicate that mental illness causes the most disability adjusted life years(DALYs) (11). A primary global concern in health care is the QoL of people with mental illnesses (12), which has gained attention in the last two decades (13). QoL is understood as a multidimensional concept that consists of four broad domains: physical, psychological, social, and environmental functioning (14). Lower perceived QoL is a challenge for those with mental illness (15–18), and those with mental illness report poorer QoL than those with other medical illness (19). Poor QoL is characterized by feelings of anguish, inadequate control, lack of self-confidence and self-esteem, sensation that one is not part of society, decreased activity and feeling of helplessness and demoralization (20). Mental illness has been found to impact social and occupational activities and prospects, drug adherence, relapse rates, and other health problems (21).

Recent studies have showed that people with mental illness have poor QoL and long-term functional impairments. For example in Brazil 67% (22), in South Africa 47% (23) and in Germany 57% (24) report poor QoL. In Ethiopia the prevalence of poor QoL is 41%, 43%, 39%, and 42% for physical, psychological, social, and environmental health domains, respectively (25). Researchers have reported on factors that contribute to poor QoL among people with mental illness. These include: sociodemographic factors such as age, sex, relationship status, education level and living condition of patients (26–31), duration of illness (28), social support (24, 32), the severity of illness (33), type of diagnosis (34, 35), the onset of illness (36) and substance use (37).

As observed above, at a prevalence of 0.48% in Ethiopia has a significant prevalence of mental illness. Given the potential impact of mental illness on QoL, studies are needed to understand how socio-ecological factors contribute to poor QoL among people with mental illness in the Ethiopian context. Structural equation modelling is a multivariate statistical analysis technique used to analyse structural relationships. This method uses factor analysis and multiple regression analysis to examine the structural relation between measured variables and latent constructs. To our knowledge, no prior research has created or validated a thorough structural model of the interactions between the numerous factors that can impact the QoL of individuals with mental illness in Ethiopia. Therefore, this study aimed to examine the QoL among people with mental illness attending psychiatry outpatient clinic and its association with sociodemographic, clinical, social support, and substance use related factors.

## Methods

### Study area

The study area is the Amhara region in Northwest Ethiopia. The Amhara region is one of the most densely populated areas in Ethiopia. The study was conducted at the University of Gondar Comprehensive Specialized Hospital in Central Gondar Zone, Ethiopia, which is 738 km Northwest of the capital city of Addis Ababa. Gondar town has a total population of 395,000 in 2022 (38).

### Study design

An institution-based cross-sectional study was conducted. An interviewer administered questionnaire was used to collect the data.

### Study population

The study population were people with mental illness (eighteen years and above) who were followed as outpatients at the University of Gondar comprehensive specialized hospital during the data collection period. People who had been followed up for at least three months for mental illness in an outpatient clinic were included. Patients with acute mental illness who could not communicate because of severe physical or mental illness during data collection were excluded during the survey.

### Sample size determination

For research using the Structural Equation Modelling (SEM) analysis approach, sample size estimation does not use a single formula. Considering this, the minimum sample size for SEM is 200, with ten observations for each observed variable and 20 cases for each estimated parameter (39, 40). However, this can’t be used in all circumstances; therefore, we decided to use a formula that can provide a maximum sample size to compute estimates. Single population proportion formula using a 95% confidence level and 4% margin of error, a 10% nonresponse rate (25), and considering a previous QoL study in Ethiopia, the term 41 % of people with mental illness have poor QoL (25). Applying the formula: n = (Zα/2)2 * P (1-P)/d2, where n is the minimum sample size required; Z is a standard normal distribution (Z=1.96) with a confidence interval of 95% and α = 0.05., d is the absolute precision or tolerable margin of error (4%), P = estimated proportion is assumed as 41% (0.41), Then n= (1.96)^2^ *(0.41) *(0.59)/(0.04)^2^= 580, adding 10% non-response rates (580 *10/100) =58, 580 + 58 = 638

### Sampling technique

The sampling interval was decided by dividing the total study participants (2400) who had a follow-up appointment during the data collection period by the total sample size 638, which gave 3.7. We reduced this to 3 because if we had rounded up to 4, we would not have achieved the desired sample size. The starting point was selected by lottery method. A maximum sample size of 638 respondents were invited for an interview but two participants refused at the middle of interview, with final sample of 636. Data were collected by psychiatric nurses with pretested interviewer-administered questionnaires.

### Data measurement tools

Validated measures were used for a number of the variables, with the remaining variables measured using single item measures.

#### Quality of life

Data on QoL was collected through interviews using the World Health Organization Quality of Life Brief. The World Health Organization Quality of Life Brief (WHOQOL-BRFE) was developed by the WHOQOL group and is a cross-culturally validated tool used to assess the patient’s QoL. We have used the Ethiopian validated Version of WHOQOL-HIV-BREF-Eth with good acceptability and psychometric properties (41). The WHOQOL-Brief includes 26 items measuring the following domains: overall perception of QoL and general health (2 items) for perceived QoL and self-rated health satisfaction, the physical health domain (7 items), the psychological health domain (6 items), the social relationships domain (3 items), and the environmental health domain (8 items). Each question is graded on a five-point scale, scoring 1 to 5. Each domain score is scaled positively (a higher score corresponds to better QoL, and QoL raw scores are transformed into a range between (0-100). The higher the mean score, the better the QoL; the lower the mean score, the poorer the QoL (42).

#### Self-esteem

Self-esteem was assessed using the single-item self-esteem scale which was created as a substitute for the Rosenberg Self-Esteem Scale. The single-item self-esteem scale is a measure of overall self-esteem. Participants rate the single self-esteem measure on five point Likert scale ranging from 1 (not very true of me) to 5 (very true of me). Despite being shortened, the scale has convergent solid validity with the Rosenberg Self-Esteem Scale and similar predictive validity (43).

#### Medication adherence

The medication adherence scale (MARS-5) assesses patients standard treatment adherence through five questions and five level response formats (1=always, 2=often, 3=occasionally, 4=rarely, and 5=never). Responses are added for a total score ranging from 5 to 25, with higher scores indicating greater adherence. Using the MARS-5 at a cut-off point greater than or equal to 20 (44, 45).

#### Substance use

Respondents who used certain substances (alcohol, khat, smoking and cannabis) for nonmedical purposes in the last year before data collection were considered current substance users (46).

#### The severity of illness

The severity of illness was measured using the Clinical Global Impression (CGI) scale of subjective and objective measurement. The CGI scale has seven responses, with responses 1-3 indicating mild, four indicating moderate, and 5-7 indicating severe.

### Data processing, building models, and analysis

**Figure 1:**
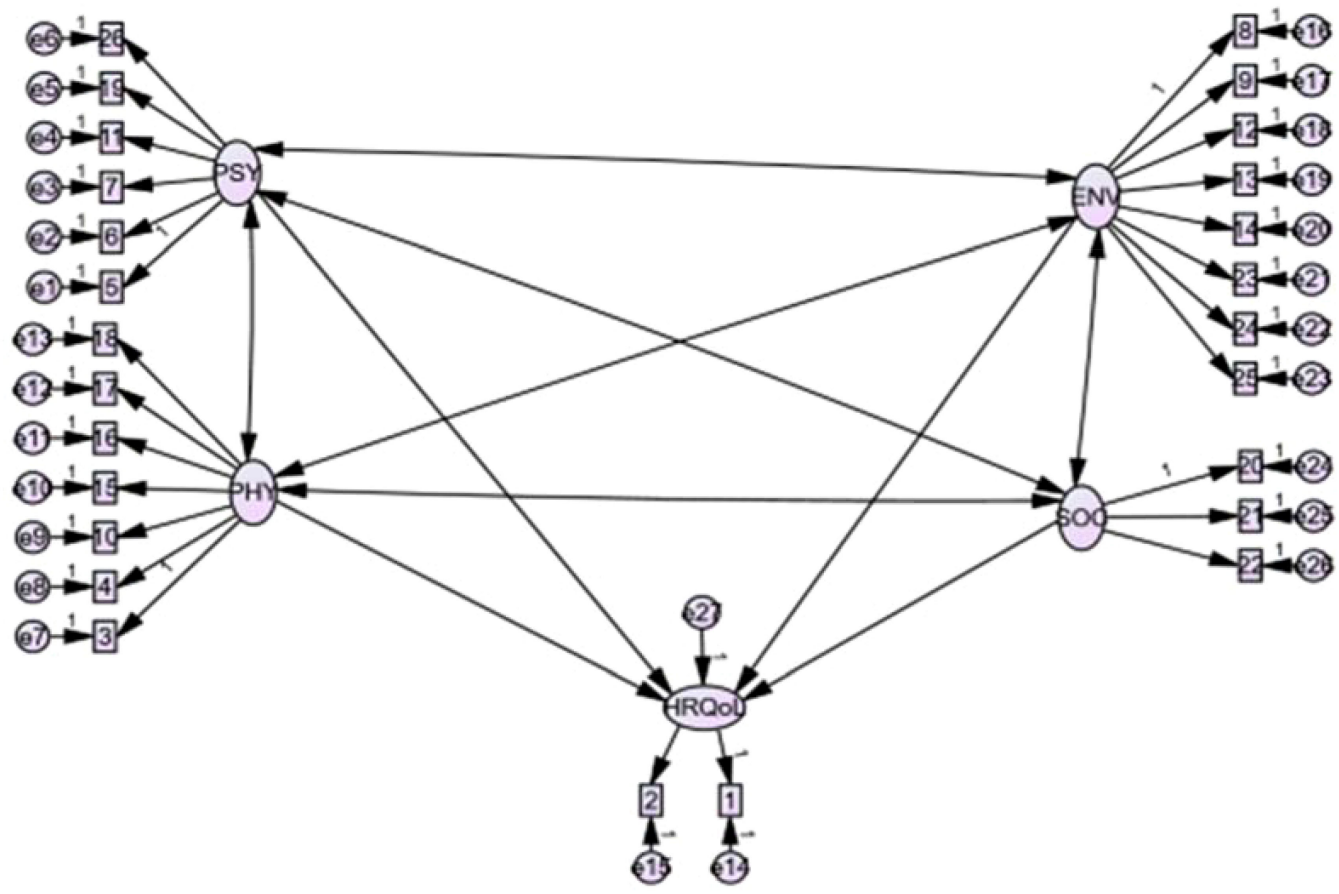
The theoretical model of health-related Quality of Life WHOHRQOL-Brief developed from different works of literature. Where: HRQoL: Health-related quality of life; PHY: Physical health domain; PSY: psychological health domain; ENV: environmental health domain; SOC: social relation health domain; Q1: overall QoL, Q2: overall health; Q3: Pain and discomfort; Q4:Medical treatment dependence; Q5: Energy and fatigue; Q6: Mobility; Q7: Sleep and rest; Q8: Daily activity; Q9: Working capacity; Q10: Positive feeling: Q11: Spirituality/personal beliefs; Q12: Memory and concentration; Q13: Bodily image and appearance; Q14: Self-esteem; Q15: Negative feelings; Q16: Personal relationships; Q17: Sex; Q18: Social support, Q19: Physical safety and security; Q20: Physical environment; Q21, financial resources; Q22: Information and skills; Q23: Recreation and leisure; Q24: Home environment; Q25: Health accessibility and quality; Q26: Transport.

Data were entered into the SPSS-28 program for analysis. The main outcome of this analysis is QoL, which is measured as a latent variable. Then structural equation modelling with maximum likelihood estimation was used to examine the relationship between various exogenous and endogenous or mediated variables. The SEM was made up of two parts: the measurement model and the structural model. The measurement components evaluate the relationship between a latent variable and indicators/items, whereas the structural components primarily indicate the relationship between the latent variables. It also provided causality between the system’s dependent and independent variables (47). To check the internal consistency of the tool, Cronbach’s α was analysed for each domain of WHOQOL-BREF. The Cronbach’s α coefficient values of 0.7 or higher were considered satisfactory (48). The WHOQOL-BREF score for each domain was calculated by averaging the corresponding items for each participant (49). The hypothesized model served as the basis for the analysis (**Figure 1**), and changes were made iteratively by incorporating mediator-independent variables or adding path relationships. If theoretically supported and comparing by Root Mean Error Approximation (RMSEA) as the absolute measure of the model fitness index and by information criteria as the measure of model parsimony. To evaluate the goodness of fit of the provided model, the Root Mean Square Error of Approximation (RMSEA) and Comparative Fit Index (CFI) were measured. Finally, an overidentified model with CFI ≥0.90 and RMSEA < 0.08 were kept. A p-value of < 0.05 and a 95% CI were used to evaluate statistical significance.

### Ethical consideration

Ethical clearance was secured from the Flinders University Human Research Ethics Committee and the Institutional Review Board of the University of Gondar. Written informed consent was obtained from people who participated in the study. Respondents were briefed and made aware of the study goal and informed of their unrestricted freedom to withdraw from the study at any time. Additionally, code numbers rather than personal identification were employed to ensure confidentiality.

## Results

### Participants sociodemographic and mental illness information

Among 638 patients invited for interviewer-administered questionnaires, 636 (99.7%) patients completed the questionnaires, with the two respondents who did not complete the questionnaires excluded from the final analysis. Around half of participants (50.9%) were female. The participants’ mean age was 35.5 years, with a standard deviation (Sd) of 11.7 years. More than three-quarters, or 497 (78.1%), of the study participants were orthodox Christians. Of the study population, 249 (39.8%) were married. Most (541, or 85.1%) lived with their immediate Family. Almost 71% (451, 70.9%) were literate and around two-thirds (431, 67.8%) were urban residents (**Tables 1**, **2**).

**Table 1:** Sociodemographic and clinical characteristics of people with mental illness in an outpatient clinic in Ethiopia, 2023(n =636).

Variables	Categories	Frequency (n= 636)	Percent (%)
Age	Mean ± Sd	35.5 ± 11.7	
Sex	Male Female	317 319	49.8 50.2
Religion	Orthodox Protestant Muslim Other	497 21 115 03	78.1 3.3 18.1 0.5
Marital status	Married Not married	253 383	39.8 60.2
Living condition	Living with family Living alone	541 95	85.1 14.9
Level of education	Literate Illiterate	451 185	70.9 29.1
Job of participant	Employed Not employed	89 547	14.0 86.0
Residence	Rural Urban	205 431	32.2 67.8
Mental illness	Schizophrenia Other MI	274 362	43.1 56.9
Age of onset of illness	</= 25yrs. >25yrs.	287 349	45.1 54.9
Duration of illness	>5 years 6month-5years	235 401	36.9 63.1
No, of episode/yr.	No episode Episodic	298 338	46.9 53.1
Hospital admission	Yes No	209 427	32.9 67.1
No Admission	No admission Admitted	428 208	67.3 32.7
Comorbid illness	Yes No	77 559	12.1 87.9
Type of drug	Antipsychotic Other drugs	301 335	47.3 52.7
Drug side effect	Yes No	163 473	25.6 74.4
Counselling	Yes No	128 508	20.1 79.9
Duration of Rx.	</=5 years >5 years	437 199	68.7 31.3
Relapse	Yes No	412 224	64.8 35.2
Suicidal ideation	Yes No	250 386	39.3 60.7
Suicidal attempt	Yes No	126 510	19.8 80.2
Family Hx. MI	Yes No	144 492	22.6 77.4
Family Hx. Subs.	Yes No	115 521	18.1 81.9
Family Hx. Suicide attempt	Yes No	29 607	4.6 95.4
waiting time in clinics	30 minute- 1hrs. 2hrs.-3hrs.	534 102	84.0 16.0
Family participates in the treatment	Yes No	536 100	84.3 15.7
Legal issues	Yes No	27 609	4.2 95.8
Objective severity	Mild Moderate and severe	493 143	77.5 22.5
Subjective Severity	Mild Moderate and sever	424 212	66.7 33.3
Tobacco Use	Yes No	43 593	6.8 93.2
Alcohol Use	Yes No	141 495	22.2 77.8
Khat use	Yes No	78 558	12.3 87.7
Cannabis Use	Yes No	04 632	0.6 99.4
Self-esteem	Low self- esteem High self-esteem	316 320	49.7 50.3
Drug adherence	Poor adherence Good adherence	87 549	13.7 86.3

Were, MI, mental illness; Hx, history.

**Table 2:** Type of mental illness in psychiatry outpatient follow-up at University of Gondar Hospital, Ethiopia, 2023(n=636).

Mental illness diagnosis of patients	Total participants	Percent (%)
Schizophrenia	274	43.1%
Depressive disorder	192	30.2%
Bipolar disorder	50	7.9%
Anxiety disorder	39	6.1%
Other psychotic disorder	68	10.7%
Stress/trauma related disorder	06	0.9%
Somatisation disorder	07	1.1%

### Self-reported health related quality of life and perceived health satisfaction

Less than a third of those who participated (22.3%) indicated that their HRQoL was good, with 204 (32.1%) reporting poor HRQoL (**Table 3**). Regarding their perceived health satisfaction, 162 (25.5%) were dissatisfied and 134 (21.1%) were satisfied, with almost half of the respondents 317 (49.8%) neither satisfied nor dissatisfied (**Table 4**).

**Table 3:** Perceived self-rated HRQoL of patients with mental illness on psychiatry outpatient follow-up at University of Gondar Hospital, Ethiopia, 2023(n=636).

Perceived self-rated QoL	Total participants	Percent (%)
Very poor	11	1.7
Poor	204	32.1
Neither poor nor good	254	39.9
Good	142	22.3
Very good	25	3.9

**Table 4:** Self-rated health satisfaction of patients with mental illness on psychiatry outpatient follow-up at University of Gondar hospital, Ethiopia, 2023(n=636).

Self-rated health satisfaction	Total participants	Percent (%)
Very dissatisfied	12	1.9
Dissatisfied	162	25.5
Neither satisfied nor dissatisfied	317	49.8
satisfied	134	21.1
Very satisfied	11	1.7

### Internal consistency and correlations between the WHOQOL-BREF domains

In order to assess the reliability of the instrument, it is necessary to evaluate its internal consistency. Cronbach’s alpha was computed for each domain. As noted, all WHOQOL-BREF domains exhibited strong Cronbach’s alpha values (α *>* 0.7). When we see factor loadings on each exogenous observed variable, components of psychological health domains had higher measurement values than other domains. However, all components of domains are statistically significant with p<0.001. The results of the inter domain correlation revealed a statistically significant association between the domains. All the associations between domains were positive. The domains of social relation health domain and physical health domain were significantly correlated with one another (r=0.92, P<0.001), psychological health domain with environmental health domain (r=0.88, p<0.001), physical health domain with psychological health domain(r=0.93, p<0.001), social relation health domain with environmental health domain(r=0.79,p<0.001), physical health domain with environmental health domain(r=0.80, p<0.001), psychological health domain with social relation health domain(r=0.86, p<0.001) respectively.

### Overall QoL and domains

There were variations in QoL domains for patients with mental illness. The highest was found in the physical health domain (50.67 ± 9.5) and the lowest in the psychological health domain (48.41 ± 10) (**Table 5**).

**Table 5:** QoL descriptive results among patients with mental illness in outpatient clinic university of Gondar hospital, Ethiopia (n = 636), 2023.

Domain	Number	Minimum	Maximum	Mean ± Sd	95% CI
Physical Health	636	25	82	50.67 ± 9.5	(49.69,51.48)
Psychological Health	636	21	83	48.41 ± 10	(47.64,49.25)
Social Relation Health	636	0.0	100	49.5 ± 18	(48.18,50.96)
Environmental Health	636	9	97	49.9 ± 15.6	(48.68,51.11)
Mean HRQoL	636	25	84	49.6 ± 10	(48.86,50.43)

Were, Sd: Standard deviation.

### The goodness of fit of the theoretical model

The following are the findings from the analysis of the hypothetical structural equation model built using exogeneous, endogenous and mediator variables: Goodness of fit (GFI)=0.91, RMSEA = 0.054, NFI = 0.86, CFI = 0.90, TLI = 0.88, AGFI=0.88 and PCMIN/DF=2.7. These indices all satisfied the recommended levels.

### Factors associated with health-related quality of life among patients with mental illness in an outpatient clinic

**Figure 2:**
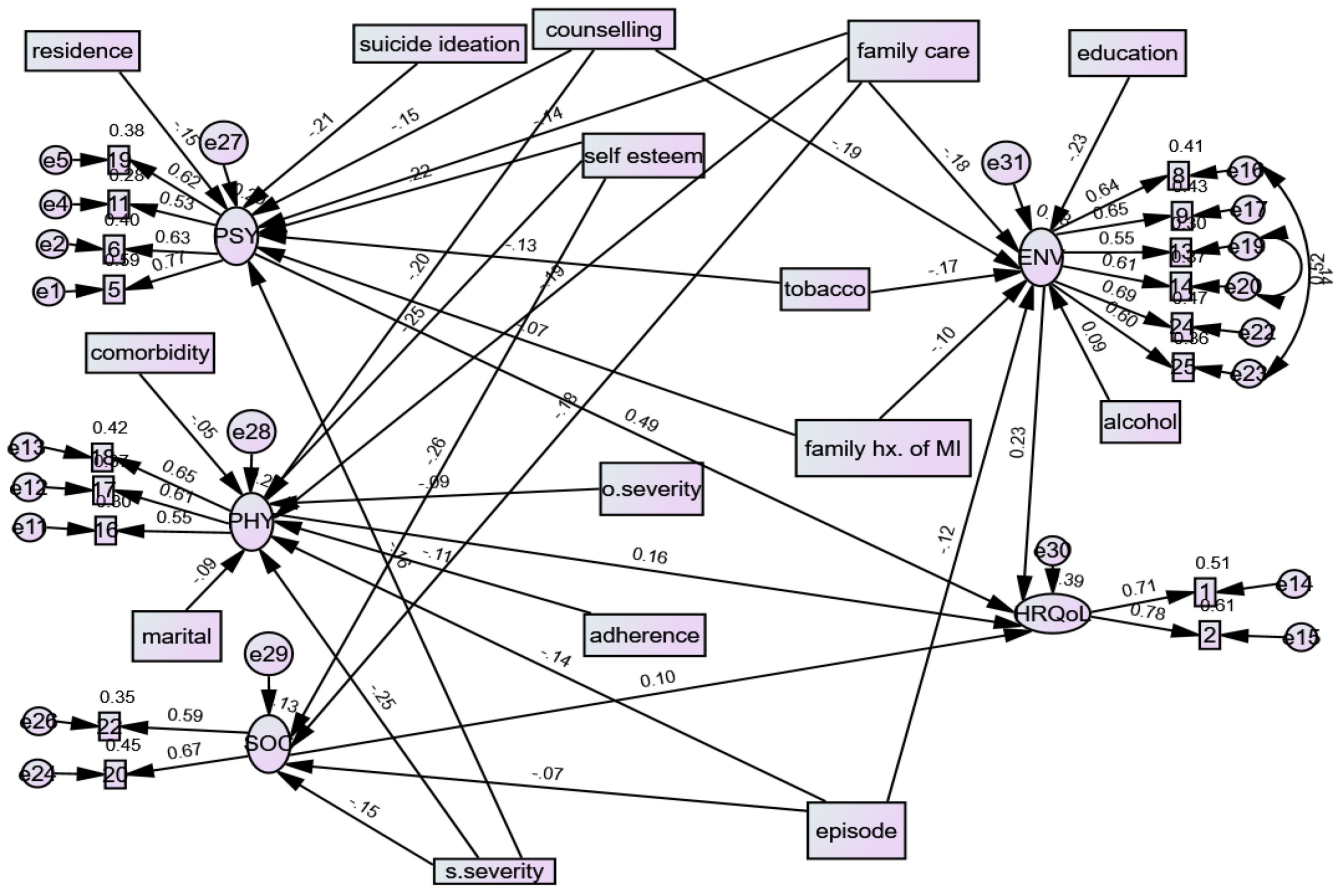
Casual factors for Quality of life among patients with mental illness in an outpatient clinic, Ethiopia, derived from the SEM, 2023.

The whole model, which combines the structural component (relationships between latent or observed variables) and the measurement component (relationships between a latent variable and its indicators or items), is displayed in (**Table 5**, **Figure 2**). Generally, the fitted model was relatively parsimonious and well-fitted with RMSEA = 0.054 and CFI = 0.90.

All the path coefficients in the diagram’s final model were statistically significant at an alpha level 0.05. The final model includes exogenous observed variables from sociodemographic factors (age, residence, educational status, and marital status), social support-related factors (self-esteem, family not participating in patient care), drug use factors (alcohol use, tobacco use) and clinical factors (objective severity of illness, subjective severity of illness, not getting counselling, suicidal ideation, number of episodes, comorbid illness, family history of mental illness), one endogenous latent variable (HRQoL), four mediator latent variables (the four domains), and 26 endogenous observed variables (QoL items).

The exogenous variables such as sociodemographic factors, social support-related factors, drug use factors, and clinical factors were directly and indirectly related to HRQoL through the mediator variables of physical health domain, psychological health domain, social relation health domain, and environmental health domains.

The structural equation model indicates that among the QoL domains, psychological and physical health domain factors had the most substantial effect on QoL, which was more significant than the causal effect of environmental and social relationship health factors.

### Casual factors of direct, indirect, and total effects of structural equation analysis

Data from this study showed that exogenous variables of clinical factors including subjective severity of illness (adjusted β=-0.250, 95% CI;-0.313, -0.120), objective severity of illness (adjusted β =-0.094, 95% CI;-0.280, -0.024), episodic illness (adjusted β-0.140, 95 CI;-0.190, -0.030), having comorbid physical illness (adjusted β= -0.050,95%CI;-0.224, -0.007), not getting counselling (adjusted β=-0.200, 95% CI;-0.298, -0.079), social support related factors like low self-esteem (adjusted β=-0.257, 95% CI;-0.365, -0.170), family does not participate in patient care (adjusted β=-0.190, 95% CI;-0.399, -0.142) and marital status not married (adjusted β= -0.090, 95% CI;-0.216, -0.036), poor drug adherence (adjusted β=-0.110, 95% CI;-0.270, -0.012) had direct negative effect on QoL through the mediator variable physical health domain. In addition, objective severity of illness (adjusted β= -0.013, 95% CI; -0.038, -0.001) and self-esteem (adjusted β=-0.011, 95% CI; -0.032, -0.001) indirect negatively affected the QoL through the mediator variable physical health domain. In addition, objective severity of illness (adjusted β=-0.107,95%CI; -0.224, -0.034) and self-esteem (adjusted β=-0.268, 95% CI; -0.377, -0.179) had a total direct negative effect on QoL through the mediator variable physical health domain.

Similarly, sociodemographic factors (rural residence (adjusted β=-0.157, 95% CI; -0.295, -0.073), clinical factors (subjective severity of illness (adjusted β= -0.160, 95% CI; -0.384, -0.159), having suicidal ideation (adjusted β=-0.217,95%CI;-0.434, -0.211), not getting counselling (adjusted β=-0.151,95% CI; -0.277, -0.006), social support related factors (low self-esteem (adjusted β=-0.140, 95% CI;-0.402,-0.136), family does not participate in patient care (adjusted β=-0.140,95%CI;-0.471, -0.131) and tobacco use (adjusted β=-0.130, 95% CI;-0.456, -0.059), and mental illness in the family (adjusted β=-0.070, 95%CI;-0.211,-0.008) had a direct negative effect on QoL through the mediator variable psychological health domain. In the same way, self-esteem (adjusted β= -0.011,95%CI; -0.030,0.001), mental illness in the family (adjusted β=-0.043, 95% CI; -0.083, -0.014) had an indirect negative effect on the HRQoL through the mediator variable psychological health domain. In addition, self-esteem (adjusted β=-0.151, 95% CI; -0.413, -0.120) and mental illness in family (adjusted β=-0.113, 95% CI; -0.259, -0.028) had total indirect negative effect on QoL through the mediator variable psychological health domain.

Likewise, clinical factors (subjective severity of illness (β=-0.150, 95% CI; -0.415,-0.105), number of episode (adjusted β=-0.079, 95%CI; -0.274, -0.077) and social support factors (low self-esteem (β=-0.260, 95% CI;-0.356,-0.105) and family not participating in patient care (β=-0.180, 95%CI;-0.433,-0.103), had a direct negative effect on QoL through the mediator variable social relation health domain.

Additionally, clinical factors (episodic illness (β= -0.120, 95% CI; -0.192, -0.028)) and not getting counselling (β=-0.190,95%CI; -0.377, -0.100), substance use factors (tobacco use (β =-0.179, 95% CI; -0.591,-0.176)), educational status illiterate (β=-0.230, 95%CI;-0.292, -0.160), and family does not participate in patient care (β=-0.180, 95% CI;-0.372,-0.073) and having a mental illness in the family (β=-0.107, 95% CI;-0.216, -0.021) had a direct negative effect on QoL through mediator variable environmental health domain. However, alcohol use (β =0.090, 95% CI;0.018,0.245) had a direct positive effect on QoL through the mediator variable environmental health domain.

In addition, the mediator variables of psychological health domain (β=0.529, 95%CI; 0.143,0.918) had a direct positive effect on QoL.

Sociodemographic factors (rural residence (β=-0.093,95%CI;-0.211, -0.020), clinical factors (suicidal ideation (β=-0.167, 95%CI;-0.293,-0.040), poor drug adherence(β=-0.091,95%CI;-0.194, -0.002), not getting counselling (β=-0.135,95%CI;-0.263, -0.016), subjective severity of illness (β=-0.180,95%CI;-0.274, -0.103), mental illness in family (β=-0.089,95%CI;-0.200, -0.010)), low self-esteem(β=-0.208,95%CI;-0.314, -0.117)) and social support factors (family not participate in care(β=-0.245,95%CI;-0.380, -0.116)) had an indirect negative effect on HRQoL (**Figure 2**, **Table 6**).

**Table 6:** The direct, indirect, and total effect of socio-demographic and clinical factors on HRQoL domains among people with mental illness.

Characteristics	Direct Effect (95%CI)	Indirect effect (95% CI)	Total Effect (95% CI)
DV: Physical health			
Subjective severity of illness			
Mild	0 0		
Moderate &severe	-0.250(-0.313, -0.120)		
Objective severity of illness			
Mild	0 0		0 0
Moderate &severe	-0.094(-0.280, -0.024)	-0.013(-0.038, -0.001)	-0.107(-0.224, -0.034)
Number of episodes			
No episode	0 0		
Episodic	-0.140(-0.190, -0.030)		
Marital status			
Married	0 0		
Not married	-0.090(-0.216, -0.036)		
Comorbid physical illness			
No	0 0		
Yes	-0.0.050(-0.224, -0.007)		
Counselling			
Yes	0 0		
No	-0.200(-0.298, -0.079)		
Self-esteem			
High self-esteem	0 0		0 0
Low self-esteem	-0.257(-0.365, -0.170)	-0.011(-0.032, -0.001)	-0.268(-0.377, -0.179)
Family participates in the care			
Yes	0 0		
No	-0.190(-0.399, -0.142)		
Drug Adherence			
Good	0 0		
Poor	-0.110(-0.270, -0.012)		
DV: Psychological			
Residence			
Urban	0 0		
Rural	-0.157(-0.295, -0.073)		
Subjective severity of illness			
Mild	0 0		
Moderate &sever	-0.160(-0.384, -0.159)		
Suicidal ideation			
No	0 0		
Yes	-0.217(-0.434, -0.211)		
Tobacco use			
No	0 0		
Yes	-0.130(-0.456, -0.059)		
Self-esteem			
High self-esteem	0 0		0 0
Low self-esteem	-0.140(-0.402, -0.136)	-0.011(-0.030, -0.001)	-0.151(-0.413, -0.120)
Counselling			
Yes	-0.151(-0.277, -0.006)		
No	0 0		
Family participates in the care			
Yes	0 0		
No	-0.140(-0.471, -0.131)		
Mental illness Hx. in the family			
No	0 0		0 0
Yes	-0.070(-0.211, -0.008)	-0.043(-0.083, -0.014)	-0.113(-0.259, -0.028)
DV: Social relation			
Number of episodes			
No episode	0 0		
Episodic	-0.079(-0.274, -0.077)		
Self-esteem			
High Self-esteem	0 0		
Low Self-esteem	-0.260(-0.356, -0.105)		
family participates in the care			
Yes	0 0		
No	-0.180(-0.433, -0.103)		
Subjective severity of illness			
Mild	0 0		
Moderate and severe	-0.150(-0.415, -0.105)		
DV: Environmental			
Number of episodes			
Not episodic	0 0		
Episodic	-0.120(-0.192, -0.028)		
Educational status			
Literate	0 0		
Illiterate	-0.230(-0.292, -0.160)		
Alcohol use			
No	0 0		
Yes	0.090(0.018,0.245)		
Tobacco use			
No	0 0		
Yes	-0.1796(-0.591(-0.176)		
Counselling			
Yes	0 0		
No	-0.190(-0.377, -0.100)		
family participates in the care			
Yes	0 0		
No	-0.180(-0.372,0.073)		
Mental illness Hx. in the family			
No	0 0		
Yes	-0.107(-0.216, -0.021)		
DV: HRQoL			
PSY domain	0.529(0.143,0.918)		
Residence			
Urban		0 0	
Rural		-0.093(-0.211, -0.020)	
Suicidal ideation			
No		0 0	
Yes		-0.167(-0.293, -0.040)	
Drug Adherence			
Good		0 0	
Poor		-0.091(-0.194, -0.002)	
Counselling			
Yes		0 0	
No		-0.135(-0.263, -0.016)	
Subjective severity of illness			
Mild Moderate & sever		0 0	
		-0.180(-0.274, -0.103)	
Self-esteem			
High Self-esteem		0 0	
Low Self-esteem		-0.208(-0.314, -0.117)	
family participates in the care			
Yes		0 0	
No		-0.245(-0.380, -0.116	
Mental illness in the family			
No		0 0	
Yes		-0.089(-0.200, -0.010)	

Hx, history.

## Discussion

The primary objective of this study was to assess the QoL and its determinants among patients with mental illness in outpatient clinic follow-up in Ethiopia. QoL outcomes among patients with mental illness are likely related to risk and protective factors at various socio-ecological levels, including individual, interpersonal, institutional, and community factors. However, unobserved variables have yet to be studied in research conducted in Sub-Saharan countries, including Ethiopia. We developed a theoretical SEM model to examine the impact of demographic factors, clinically related factors, social support-related factors, and substance use-related factors on QoL and then assessed the model’s goodness of fit and the significance of the direct and indirect paths.

This study examined the mean scores for each domain of QoL and overall HRQoL for patients with mental illness in outpatient clinics. Patients with mental illness receiving follow-up care in an outpatient clinic showed poor QoL across all domains (mean QoL= 49.6 ± 10). These results showed that the psychological health domain was more affected than others, with a mean HRQoL of 48.41 ± 10. This finding was supported by previous studies conducted in Ethiopia that showed poor QoL amongst adult patients with common mental disorders attending an outpatient clinic, and among chronic illness patients and youth with substance users (50, 51). The psychological health domain has been reported to be more affected by mental illness because this has a particular impact on completing many everyday duties, limiting level of independence and contributing to low self-confidence and self-esteem (52, 53).

According to our findings, 32% of participants had perceived poor HRQoL and almost 40% of these populations perceived had neither poor nor good HRQoL (**Table 4**). The poor HRQoL was likely related to the way that mental illness affects social, cognitive, and active functioning, which might affect the adoption of health behaviors such as poor sleeping patterns, smoking, or abuse of drugs, a vicious cycle resulting in worsening QoL (54).

The study also outlines the determinant factors of QoL finding a complex web of factors that were associated with QoL. In terms of sociodemographic factors, the results indicate that being illiterate and having low educational status was associated with poorer QoL in the environmental health domain. This result was inconsistent with research conducted in Ethiopia among patients with schizophrenia, where having no formal education was negatively associated with the physical health domain of QoL (55). This could be supported by the fact that improved mental health is related to higher educational level. However, studies have shown that education is also a predictor of employment, wealth, and social standing which have been associated with QoL; as a result, it has a high degree of predictive value for better health and well-being (56, 57).

Marital status had a significant relationship in the physical health domain. Not married patients had a negative direct association with the physical health domain. This finding is in line with a study conducted in Ethiopia that found that being divorced was inversely associated with QoL for those with mental illness (55). In Korea, the results of a multilevel analysis by marital status revealed that single men had significantly poorer QoL than married men. However, this study was contrary to study conducted in southern Ethiopia which showed, compared to married people, those with mental illness who were single had better QoL (58). This may be because marital status is an essential socioeconomic variable linked to life span and good health, with many studies findings that married persons had better health outcomes than their single counterparts (59–61).

Similar to the previous study conducted in Ethiopia (50), the current study showed that rural residence had a direct negative relationship with QoL through the mediator variable psychological health domain and an indirect negative relationship with overall HRQoL. A previous study has found that being a rural resident was negatively associated with physical health domain among schizophrenia patients in Ethiopia (55). Being from a rural residence could pose a risk factor for developing mental health disorders due to poverty, resource shortage, and lack of educational opportunities. In general, rural communities have lower median household incomes, employment rates, and levels of education than the overall population which may contribute to poorer QoL (62).

A number of clinical factors were also identified as influencing QoL. Using this structural model, we found that the subjective severity of illness, being moderate and severe, was negatively associated with poor QoL in physical and psychological health domains. The objective severity of illness being moderate and severe was also negatively associated with the physical health domain, social relation health domain, and overall QoL compared to those with mild severity. This study finding concurs with previous research conducted in Germany that found an association between severity of illness and poor QoL (52). This could be linked with expectations for performing a job function being affected by severity and greater severity more likely to affect social relationships.

This study revealed an inverse association between episodic illness and QoL. When the number of episodes of mental illness increases, it affects the QoL of patients negatively directly in the physical health domain and environmental health domain. Similar findings were found in a study conducted in Germany on patients with severe mental illness in outpatient care (52). This might be explained by episodic illness, which may have frequent relapses affecting the QoL.

Previous studies have also reported that patients with suicidal ideation had a significantly poorer QoL than those without suicidal ideation (63). Our study likewise showed that suicidal ideation had a direct negative relationship with QoL through the psychological health domain and indirect negatively to overall QoL domains, respectively. This is also in line with studies conducted in Australia (64) and Korea (63).

This investigation found that having comorbid illness was inversely associated with QoL through the direct physical health domain. This is similar to a study conducted in Ethiopia and a study done in Taiwan (61). This might be explained by the fact that comorbidities cause people to become dependent on various medications and that taking multiple medications could contribute to worsening QoL because of their side effects or drug interactions with other medications.

In our study structural equation analyses revealed a statistically significant negative association between receiving counselling and QoL. This study showed that receiving counselling was inversely associated with the physical health domain, psychological health domain, environmental health domain and indirectly with overall QoL. It may be that counselling enhances coping skills, boosts confidence, lowers anxiety, increases social and public functioning, and assists patients in addressing concerns associated with their mental health conditions, which impact the rest of their lives (65, 66).

The current study identified that having a family history of mental illness had a direct and an indirect negative association with the psychological health domain, a direct negative effect on environmental health domain, and a indirect negative effect on overall QoL. However, we did not find another study that shows a similar association to support our finding. QoL had direct positive relationship with the mediator variables psychological health domains but no association with health domains. This finding is supported by previous studies done in Ethiopia among common chronic disease, and youth substance users (51).

In the current study, having poor drug adherence was significantly associated with the physical health domain and overall QoL. Poor drug adherence had a direct negative association with the physical health domain and indirect negative association with overall QoL. This finding was supported by previous study conducted in southwest Ethiopia where medication non-adherence was associated with lower mean scores of quality of life and lower level of satisfaction in social relation domain, and medication non-adherence was independently associated with lower QoL in diabetic patients in Greece (67). This may be related to poor drug adherence having a negative impact on the course of illness resulting in relapse, frequent hospitalization, long time to remission, and suicide intention (68).

In terms of substance use factors, our findings showed that alcohol use had direct positive association with the environmental health domain. This outcome is consistent with a systematic review of research studies conducted in different settings that found that those using alcohol were less likely to have good QoL (69). For example, in a community based study in India, alcohol users had poorer QoL than non-users (70). There is a growing body of research that has demonstrated that alcohol use lowers QoL (71, 72), particularly in the emotional health domain (73, 74), mental health domain (75, 76), and social relation health domains (77).

The study also found that tobacco use was associated with worse QoL, with a direct negative relationship with psychological and environmental health domains. This supports other studies conducted in different populations where tobacco use was associated with a reduced probability of having a higher QoL (78–80). Smoking tobacco increases the chance of developing chronic medical illnesses such as chronic obstructive pulmonary disease, cardiovascular disorders, and cancer, which can worsen QoL (79, 81).

The study also investigated associations between QoL and social support factors. The relationship between self-esteem and QoL for people with mental illnesses has been investigated previously. Our study’s findings showed that low self-esteem had a direct and indirect negative effect on the physical health domain and indirect negative effect on overall QoL. This is consistent with a study done in North India on bipolar patients and a negative relationship between QoL and self-esteem in Singapore on psychiatry patients (82). This could be because self-esteem is essential for one’s activities, emotional and physical fulfillment, daily life coping, and establishing and sustaining relationships (83). Additionally, in a society whose norms strongly focus on an individual’s happiness and self-image, a decrease in self-esteem might be a critical risk factor for the decline in QoL dimensions (84, 85).

In most countries, families are supposed to be the primary caretakers of patients with mental illness (86). Our study found that having family who do not participate in patient care had a negative direct effect on the physical health domain, psychological health domain, social relation health domain, and environmental health domain. It might be that if the family takes on caregiving duties, the family member would have the advantage of being familiar with the patient’s unique needs, routines, personality, and wants, which could bring some comfort to the patient, reducing the suffering and stress and improving patient QoL (87). On the other hand, not having this support could undermine QoL.

### Strengths and limitations of the study

To the best of our knowledge, this is the first study to comprehensively examine the effects of sociodemographic factors, clinical factors, substance use factors, and social support-related factors on the QoL among patients with mental illness in outpatient clinics in Ethiopia. This study applied a structural equation model for latent variables and QoL models and examined both direct and indirect pathways of association. The study also used standardized tools for data collection. Data was collected by trained and experienced psychiatry nurses and supervised by two psychiatry professionals. All ethical research procedures were carefully followed. Nevertheless, this study has some limitations. Firstly, the structural equation model only allows for binary variables as measurement components, which could result in information loss. Secondly, as the study is cross-sectional it is not possible to show a cause-and-effect relationship. Thirdly, there may be a risk of social desirability bias because the study was institution based. Fourth some of measuring tools like self-esteem, drug adherence were not validated in Ethiopia. Finally, we excluded those who are critically ill from participating in this study which means were likely underestimated the impact of mental illness on QoL given those with the largest likely impacts were excluded.

### Conclusion

This study showed that patients with mental illness in an outpatient clinic in Ethiopia experienced poor QoL, with the psychological health domain mostly affected. To mitigate the impact of poor psychological health among mentally ill patients such as these, suitable psychological health promotion programs are essential for enhancing health related QoL. The study identified sociodemographic variables (residence, educational status, marital status), social support-related factors (self-esteem, family not participating in patient care), drug use factors (alcohol use, tobacco use), and clinical factors (objective severity of illness, subjective severity of illness, not getting counselling, suicidal ideation, number of episodes, comorbid illness, family history of mental illness) that were directly or indirectly associated with QoL. These factors should receive special attention from program planners and policymakers in addressing poor QoL to patient with mental illness.

## Data availability statement

The raw data supporting the conclusions of this article will be made available by the authors, without undue reservation.

## Ethics statement

Ethical clearance was secured from the Flinders University Human Research Ethics Committee and the Institutional Review Board of the University of Gondar. Written informed consent was obtained from people who participated in the study.

## Author contributions

WA: Conceptualization, Data curation, Formal analysis, Investigation, Methodology, Project administration, Resources, Software, Supervision, Validation, Writing – original draft, Writing – review & editing. LM: Conceptualization, Data curation, Investigation, Methodology, Writing – review & editing. CD: Supervision, Writing – original draft, Writing – review & editing. TA: Supervision, Writing – original draft. AZ: Conceptualization, Data curation, Investigation, Methodology, Project administration, Resources, Supervision, Writing – original draft, Writing – review & editing.
